# Supplementary material for: The influence of sex, gender, age, and ethnicity on psychosocial factors and substance use throughout phases of the COVID-19 pandemic
Source: PLoS One. 2021 Nov 22;16(11):e0259676. doi: 10.1371/journal.pone.0259676 (PMC8608308; doi:10.1371/journal.pone.0259676)
Supplement: Attachment — Submitted filename: PONE response to reviewers_Oct 9.docx [file pone.0259676.s002.docx]

PONE-D-21-18028

The influence of sex, gender, age, and ethnicity on psychosocial factors and substance use throughout phases of the COVID-19 pandemic

**Responses to the Editor**

We have removed all blinded information and replaced it with identifying information.

We have now included this information in the Methods.

**Responses to the Reviewers**

1. **Reviewer #1:** “The influence of sex, gender, age, and ethnicity on psychosocial factors and substance use throughout phases of the COVID-19 pandemic” applies gender and intersection lenses to an investigation of mental health and substance use. It includes over 6,000 people living in Canada, the majority of whom are white (81%), heterosexual (85%), women (87%), living in an urban area (94%), who earn over $80,000 per year (57%) and have access to email as well as the internet (100% by virtue of recruitment). Authors report that outcomes are significantly associated with pandemic phase, gender, and age.

   This is an important topic and little research has been conducted in regard to the COVID-19 pandemic. The authors report interesting findings, which could help inform responses to future pandemics. Three points dampen my enthusiasm for the paper. First, sampling methods appear to be haphazard, making it difficult to understand the results and to whom they can be generalized. Second, the large number of outcomes renders the discussion as rather surface and lacking an anchor or main “thread.” Third, the novel findings discovered here aren’t highlighted in a way that makes them stand out; how they advance the field, and how they should be used need to be further developed.

   A few suggestions:

   1. The title and abstract refer to a gender lens and the introduction refers to an intersectional lens. The introduction then goes on to motivate a gender-based study, but brings back intersectionality in paragraph 5. It would be helpful for the authors to commit and focus the paper on either gender or intersectionality (which includes gender, but is a different topic and needs to be set up differently). If intersectionality is be the focus of the paper, it should be defined and the paper will need some editing. Intersectionality is generally considered the interaction between aspects of social status (gender, race, socioeconomic status), which requires a different introduction (different motivation). For example, ethnicity is a major player in intersectionality, but the authors include only one sentence on minority stress in the introduction (and as an “other social determinant of health), with nothing about how race, sex and socioeconomic status have been shown in prior research to influence outcomes studied here. Also, if the paper is focused on intersectionality, interaction effects should be the main effects of interest.

**Our Response:** We thank the Reviewer for this comment and we have focused the paper on our primary interest, which is gender. As such, we have removed mention of intersectionality in the Introduction, and strengthened our focus on gender.

2. The authors introduce a lot of topics in the introduction which makes the point of the paper unclear. It would be helpful for the authors to streamline the text a bit. Also, while a variety of topics are raised, several key topics are left out. For example, each outcome is a huge topic in itself and to understand how this study advances science, the reader needs to understand what is already known about the outcomes (e.g., why are the questions asked here important for each outcome?) Understanding what results reported here mean will require at least some background on each outcome (or background on considering them collectively). This may be a lot to accomplish for so many outcome variables.

**Our Response:** We have significantly re-written the Introduction to clarify that our interest was firstly on the impact of gender on depression, anxiety, stress, and loneliness. Secondarily we were interested in the effects of gender on alcohol and cannabis use. In addition to the direct effect of gender, we explored interactions of gender with other sociodemographic variables such as age, ethnicity, income, and other variables.

3. “Phases” of the pandemic are mentioned, but what they are and why they matter is not specified. Are the dates the authors assigned to phases arbitrary? Exact what does each phase represent? The authors hypothesize that “during phases of increased social restriction psychosocial symptoms would increase,” suggesting that social restriction somehow contributes to the definition of each phase, but exactly how that happens is unclear.

**Our Response:** We have clarified in the Introduction why we were interested in retrospectively analyzing our psychosocial our outcomes across phases of the pandemic. Specifically, we asked participants to reflect on their mental health at different phases that corresponded with different provincial stages of public health mandates pertaining to social restrictions. We have also sought to clarify this in the Methods as well.

4. More detail on inclusion criteria, sampling methods and recruitment venues of the original studies is needed.

**Our Response**: We have included more information about the original cohorts, who then received invitations to participate in the present study. We have released a report that details the original samples and provided a citation for this report [28].

5. The statistical analysis section states that longitudinal analyses were conducted, but a limitation of the study cited by authors is “cross-sectional nature of the survey, where participants completed the survey at only one time point.” Please clarify.

**Our Response:** We have clarified again that this was cross-sectional, and that participants reflected retrospectively on their self-report outcomes across the various time points. We have removed “longitudinal” from this section.

6. There are multiple types of longitudinal data analysis so please be more specific about analytic methods.

**Our Response:** Mixed-effects models with person ID as a random effect were used to allow for assessments over time. This is stated at the beginning of the statistical analysis section, but we've added a few words to clarify.

7. The abstract lists six outcomes, but the last paragraph of the introduction seems to suggest only four mental health outcomes, with analyses that consider how these outcomes are associated with the substance use variables. This may just be a matter of a few edits to the final sentences of the introduction, but please be specific and consistent throughout the manuscript to improve clarity.

**Our Response:** We thank the Reviewer for pointing this out. We have now made it clear at the end of the Introduction that the focus on psychosocial outcomes was of primary interest (i.e., depression, anxiety, stress, and loneliness), and the analyses of alcohol and cannabis use were exploratory.

8. The last paragraph of the introduction also refers to the four “psychosocial domains.” Please explain how the four mental health variables map onto psychosocial domains. Referring to a score intended to identify a mental disorder as a domain is confusing, as is some of the text around it (e.g., “We predicted that these four psychosocial domains would be more symptomatic in women and gender diverse individuals”).

**Our Response:** We have now edited the end of the Introduction to make it clear that we are interested in the symptoms of depression, anxiety, stress, and loneliness, and have removed the confusing reference to “psychosocial domains”. We have removed any mention of “domain” in the manuscript.

9. Given that potential participants were invited to enroll in the current study via email and to take an online survey, people without email or access to the internet were left out. This overlooked low-income individuals, which is a limitation of the study and should be cited as such.

**Our Response:** We have added to the Limitations section of the Discussion that only participants with access to email, and means to complete the survey online participated.

10. Authors state “To improve the representativeness of the study sample, Index Participants were asked to pass the invitation on to one household member who identified as a different gender as the respondent.” While this would have increased the sample size, and possibly increase gender variability, it would not necessarily increase representativeness of the population. To the contrary, sampling from the same household results in another person who is very similar to the first in terms of race and socioeconomic factors. In the results section, it becomes clear that this population is in fact composed of many more women (87%) than men. Why the population is so skewed toward women deserves explanation.

**Our Response:** This is a very fair point. We have changed some wording here to clarify that this was mainly to increase gender diversity rather that overall sample representativeness. The reason for the gender skew to the data is because the original cohorts for recruitment came from studies that had predominantly enrolled women. This is stated in the Methods section.

11. The authors do not appear to have used a measure of loneliness that was previously tested for reliability and validity. It would be helpful for the authors to provide assessment of reliability and validity for the measure they created here.

**Our Response:** We used the Coronavirus Health and Impact Survey (CRISIS) to measure loneliness. CRISIS was developed early in the COVID-19 pandemic to assess mental distress and resilience, and has been validated in large samples in the UK and USA. The Mood States domain that houses the item asking about loneliness is found to have excellent internal and test retest reliability (Omega > 0.9; ICCs between 0.79-0.87), and individual items on the CRISIS showing high ICC. Construct validity was also very high by showing that the CRISIS with other validated measures of affect. The CRISIS has been suggested to be useful for population-based mental health research during the COVID-19 pandemic (Nikolaidis et al., 2021, Nature Portfolio). We have included some of this information in the Methods section of the revised paper.

12. A major problem with this study is understanding exactly who it represents. It starts out including people who have participated in prior research (but recruitment criteria are not provided). It then goes on to state that, after two months of data collection, recruitment was expanded. Why expansion occurred is unclear and how the additional sampling venues were chosen is similarly unclear. Public recruitment through social media, and “engagement of community groups and other stakeholders” (without explanation as to what is meant by stakeholder in this specific scenario) make it very difficult to understand who this study applies to and what it means. The number recruited using each sampling mode is not stated in the results, and there is also no attempt to account for recruitment mode in analyses.

**Our Response**: As described in our response to Reviewer comment #4 above, we have further described the original cohorts that were invited to participate. We have also added to the revised manuscript that the rationale for expanding recruitment to public sources two months into data collection was in order to help us meet our a priori target of n = 750 participants for each age-based strata. We have also identified the name of the Women’s Health Research Institute that was leveraged to contact a more general public audience. We have removed the ambiguous language about stakeholder groups.

13. On average, the sample seems to be college-educated, straight, white women who live in an urban environment, have access to the internet, and earn over $80,000 a year. This isn’t entirely clear and it would be helpful to distill information like this in a summarized fashion to give the reader an idea of who is included.

**Our Response:** While it is true that most are women, white, post-secondary educated, and straight, given the large sample size, we still have 750 men, 800+ non-straight, 1000+ non-white, etc. We believe that this information is clearly presented in Table 1 and don't feel that it needs to be reiterated more than it already is in the Results section to avoid repeating table and text information.

14. Regarding lines 152 and 153: how was the target sample size of 750 determined?

**Our Response**: As we indicate in the “Participant Recruitment” section of the manuscript, “Participants were stratified into nine five-year age strata, and using a SARS-CoV-2 population seroprevalence of 2% (±1, 95% CI), the target recruitment for each stratum was 750. The seroprevalence statistic was used to target recruitment for analyses in a separate manuscript.

15. Discussing mental health scores is confusing (e.g., lower usually indicates worse health, but in this case, it refers to fewer symptoms and better health). Consider referring to the number of symptoms throughout instead of scores.

**Our Response:** We have carefully revised the Results and Discussion section to ensure we are clear that higher scores means more psychosocial symptoms.

16. The text refers to symptom scores (suggesting a continuous variable), but the table simply states each mental health condition (suggesting a dichotomous variable). Given that the methods section describes both formats, this is confusing. Consider clarifying the format of the table variables on the table.

**Our Response**: Tables 2 and 3 present the results of mixed-effects linear regression models using the continuous scores of depression, anxiety, stress, and loneliness. We have also verified that the Methods section describing each of these outcomes indicates the scale ranges for each measure.

17. The text refers to sex and gender separately and I’m assuming that the headers in Table 1 also refer to gender, but it would be helpful to specify that “women” and “men” refers to gender and not sex. Given the focus on gender and sex in this paper, it would also be helpful to add a row showing how sex breaks down by gender.

**Our Response**: Paragraph 3 of the Introduction goes into detail on how sex (male, female, intersex) and gender (woman, man, transgender, gender-diverse) are defined. We have added a statement to the text to make it clear that gender groups presented in Table 1 are women, men, and gender diverse.

18. The methods section states, “We included pairwise interactions to assess non additive effects between pandemic phase, sex, gender, ethnicity, sexual orientation, income, and Indigenous status.” However, the results section leads with a series of interactions based on age. There is a disconnect here. Also, intersectionality is often thought of as being based in race and gender, so the results section was surprising due to its inconsistency with both earlier methods text (which did not emphasize age) and the field of study (which often focuses on gender and race). Age is certainly an important factor; so authors might want to consider (1) developing the introduction in a way that talks about age as a component of intersectionality, and (2) being consistent between methods and results in terms of factors discussed.

**Our Response:** This is true, however the results for the other interactions are mentioned further in the results section.

19. Unless I missed it, there were no significant interactions between race and sex or race and income or sex and income with regard to the mental health and substance use outcomes. It may be helpful to clearly state this in the discussion.

**Our Response:** This has now been stated clearly in the 4^th^ paragraph of the Discussion.

20. The discussion is rather surface and fails to highlight what is new here and how, in combination with prior research, results could be used to improve policy or practice (i.e., beyond the fact that it should be used, exactly how should it be used?) For example
●The discussion states that women were more likely to have worse mental health scores and this is consistent with prior studies. How was this study different, novel or unique; how do the novel findings contribute something new here; and how should policy or practice be altered based on this study?
●There is a paragraph devoted to why age may have been associated with study outcomes, but I wonder if the authors have any suggestions for changes to pandemic-related policy or practice based on age? Or how future research could use this finding?
● The discussion states that this study, which found no gender effect on alcohol use, is in contrast to a prior study. Why might that be? Geographic differences? Differences in the population? Differences in terms of the time point (relative to the beginning of the pandemic) studied?
● The authors state, “These findings illustrate that government policies and interventions for future pandemics should place on emphasis on young adults, low-income populations, women, Indigenous, and gender diverse communities.” I wonder what this means and how it would be done. It would be extremely helpful for the authors to cite one or two pandemic policies and suggest exactly how they could have placed an emphasis on the factors identified here.
● Similar to the prior bullet point, the final conclusion of the abstract is, “Our findings highlight the need for policy makers and leaders to proactively consider gender when tailoring public health measures for future pandemics,” which is rather generic. It could have been written (based on prior COVID research) before this study was conducted. What do these results uniquely suggest that policy makers should do specifically?

**Our Response:** Thank you for all of these suggestions, which we have now incorporated.

21. The paragraph regarding stigma against Chinese culture and validity of study measures in Chinese Canadians is long and seems to be a tangent. Consider a more succinct paragraph on how these results harmonize with prior studies and how findings could be put to practice.

**Our Response:** We have significantly stream-lined this paragraph to focus on the most pertinent findings: that Chinese participants experienced fewer psychological symptoms, and that this might be due to lower rates of disclosure, or issues pertaining to the validity of these measures.

22. Authors state that there was a trend for increasing odds of increased alcohol use as household income increased. However, given that none of the income categories were significantly different than the reference, this seems unlikely and the statement needs justification (e.g., was the trend statistically significant?)

**Our Response:** The overall p-value for income is 0.03 (which is indicated in the footnote of the table) making this a significant trend. None are different compared to the reference, but other pairwise comparisons are significant. This is not an uncommon feature of using categorical variables and reporting odds ratios with reference to a particular group.

23. The abstract states that participants were asked questions “across five phases of the pandemic as well as retrospectively before the pandemic,” which seems to suggest multiple measures. The text should be edited to clearly indicate that this is a cross-sectional study.

**Our Response:** We have edited various parts of the manuscript to make it clear that the design was cross-sectional, and that participants were asked to retrospectively report on their anxiety, depression, stress, loneliness, alcohol, and cannabis use at various time points.

24. Consider “fewer symptoms” rather than “less symptoms.”

**Our Response:** We have made this revision throughout.

25. Consider additional editing (e.g., using “have” multiple times in the same sentence; also, regarding Lines 84 and 85, rather than stating that age may interact with sex and psychosocial outcomes [suggesting a 3-way interaction], I think the authors mean that age may interact with sex to influence psychosocial outcomes).

**Our Response:** Thank you for making these editorial suggestions, which we have incorporated.

**Reviewer #2**: 

I have perused with great interest your praiseworthy contribution titled The influence of sex, gender, age, and ethnicity on psychosocial factors and substance use throughout phases of the COVID-19 pandemic, which aims to elaborate on one of the most consequential aspects of the ongoing pandemic. The article is well-written and competently assembled. The discussion is broad-ranging and thorough, its conclusions well supported by solid methodology.
Analyzing the influence of sex, gender, age and ethnicity confers an element of novelty to the report which makes it a valuable piece of research of considerable interest for the public at large. Discrepancies in how deeply the pandemic has impacted different segments of the population are well expounded upon, and that itself has value in terms of contributing to more effective policy-making approaches and strategies.

1. An element of weakness attributable to studies such as this has to do with its being cross-sectional, which makes it all but impossible to establish the actual correlations and causality directions taking into account all the study's variables. In that regard, future longitudinal studies will go a long way towards establishing actual causality. All data were self-reported, hence liable to be affected by well-established method biases, as you hinted.

**Our Response:** We have indicated in the Limitations section of the Discussion that the retrospective and cross-sectional nature of the study design was a limitation.

2. Since the title mentions "substance use", I believe it would be advisable for the authors to dig a bit deeper than alcohol and cannabis, although their research has only accounted for those.

**Our Response:** We have made it clear in the Abstract that by substance use, we specifically looked at alcohol and cannabis use. We did not explore other aspects of substance use.

3. I would recommend making a brief subchapter or mention in the conclusions as to how substance use worldwide has been profoundly reshaped as a result of the pandemic, which might dovetail with some of the conclusions you arrived at based on your data, both from a substance use perspective and a psychosocial one. That would go a long way toward broadening the scope of your article, allowing for more in-depth comparisons of your own findings versus other sources.

**Our Response:** We have added a paragraph in the Discussion section, following our previous discussion of substance use to broaden the scope of our findings.

You may want to draw upon the following sources:

Zaami S, Marinelli E, Varì MR. New Trends of Substance Abuse During COVID-19 Pandemic: An International Perspective. Front Psychiatry. 2020 Jul 16;11:700. doi: 10.3389/fpsyt.2020.00700.

Ornell F, Moura HF, Scherer JN, Pechansky F, Kessler FHP, von Diemen L. The COVID-19 pandemic and its impact on substance use: Implications for prevention and treatment. Psychiatry Res. 2020 Jul;289:113096. doi: 10.1016/j.psychres.2020.113096.

Stack E, Leichtling G, Larsen JE, Gray M, Pope J, Leahy JM, Gelberg L, Seaman A, Korthuis PT. The Impacts of COVID-19 on Mental Health, Substance Use, and Overdose Concerns of People Who Use Drugs in Rural Communities. J Addict Med. 2020 Nov 3:10.1097/ADM.0000000000000770. doi: 10.1097/ADM.0000000000000770.

Zaami S. New psychoactive substances: concerted efforts and common legislative answers for stemming a growing health hazard. Eur Rev Med Pharmacol Sci. 2019 Nov;23(22):9681-9690.


4. Lastly, since your manuscript qualifies as a research article, it would be best to have a structured abstract with Objectives, Methods and study design, Results and Conclusions.

**Our Response:** Thank you for this suggestion. We have added these subheadings to the Abstract.
